# Diazepam Impairs Innate and Adaptive Immune Responses and Ameliorates Experimental Autoimmune Encephalomyelitis

**DOI:** 10.3389/fimmu.2021.682612

**Published:** 2021-07-20

**Authors:** Cristian R. Falcón, Nicolás Fernández Hurst, Ana Laura Vivinetto, Pablo Héctor Horacio López, Adolfo Zurita, Gerardo Gatti, Laura Cervi, Clara G. Monferran, German A. Roth

**Affiliations:** ^1^ Centro de Investigaciones en Química Biológica de Córdoba (CIQUIBIC, UNC-CONICET), Departamento de Química Biológica, Facultad de Ciencias Químicas, Universidad Nacional de Córdoba, Córdoba, Argentina; ^2^ Instituto Multidisciplinario de Investigaciones Biológicas (IMIBIO-CONICET), Universidad Nacional de San Luis, San Luis, Argentina; ^3^ Instituto de Investigacion Medica Mercedes y Martin Ferreyra, INIMEC–CONICET Córdoba, Córdoba, Argentina; ^4^ Centro de Investigaciones en Bioquímica Clínica e Inmunología (CIBICI, UNC-CONICET), Departamento de Bioquímica Clínica, Facultad de Ciencias Químicas, Universidad Nacional de Córdoba, Córdoba, Argentina; ^5^ Fundación para el Progreso de la Medicina, Laboratorio de Investigación en Cáncer, Córdoba, Argentina

**Keywords:** diazepam, autoimmunity, experimental autoimmune encephalitis, immunomodulatory, inflammation

## Abstract

Currently there is increasing attention on the modulatory effects of benzodiazepines on the immune system. Here, we evaluate how Diazepam (DZ) affects both innate and adaptive immunity. We observed that treatment with DZ and Lipopolysaccharide (LPS) on macrophages or dendritic cells (DCs) induced a defective secretion of IL-12, TNF-α, IL-6 and a lesser expression of classical activation markers as NO production and CD40 in comparison with LPS condition. More importantly, mice pre-treated with DZ and then challenged to LPS induced-septic shock showed reduced death. The DZ treatment shifted the LPS-induced pro-inflammatory cytokine production of peritoneal cells (PCs) to an anti-inflammatory profile commanded by IL-10. In agreement with this, DZ treatment prevented LPS-induced DC ability to initiate allogeneic Th1 and Th17 responses *in vitro* when compared with LPS-matured DC. Since these inflammatory responses are the key in the development of the experimental autoimmune encephalomyelitis (EAE), we treated EAE mice preventively with DZ. Mice that received DZ showed amelioration of clinical signs and immunological parameters of the disease. Additionally, DZ reduced the release of IFN-*γ* and IL-17 by splenocytes from untreated sick mice *in vitro*. For this reason, we decided to treat diseased mice therapeutically with DZ when they reached the clinical score of 1. Most importantly, this treatment ameliorated clinical signs, reduced the MOG-specific inflammatory cytokine production and prevented axonal damage. Altogether, these results indicate that DZ is a potent immunomodulator capable of controlling undesired innate and adaptive immune responses, both at the beginning of these responses and also once they have started.

## Highlights

- Diazepam treatment impairs innate immune response *in vitro* and *in vivo*.
- Bone marrow-derived dendritic cells treated with LPS in the presence of Diazepam develop a defective ability to initiate adaptive inflammatory responses.
- Therapeutic treatment with Diazepam of EAE-sick mice decreases clinical signs and prevents axonal damage and demyelination.

## Introduction

Benzodiazepines are psychoactive drugs that share similar pharmacological properties, such as sedative, hypnotic (sleep-inducing), anxiolytic, and anticonvulsive action and are widely used adjuncts to anesthesia to induce central muscle relaxation and amnesia (1). In addition, these drugs also affect immunity. The effect of benzodiazepines depends on the activation of binding sites, such as the central and peripheral benzodiazepine receptors. The central benzodiazepine receptor is mainly present in the central nervous system (CNS) and forms part of the *γ*-aminobutyric acid (GABA)_A_ receptor complex (2). The peripheral benzodiazepine receptor is a ubiquitously expressed protein of the outer mitochondrial membrane termed translocator protein 18 kDa (TSPO), structurally and functionally different from the GABA_A_ receptor (3). The TSPO is expressed in platelets, immune cells, endothelium, vascular smooth muscle, bone marrow, endocrine cells and to a lesser extent in the CNS where it is associated with glial cells (4, 5). Upregulation of the TSPO is observed in many CNS diseases, and some TSPO ligands are currently under investigation as therapeutic means for promoting neuroprotection, axonal regeneration, and modulating inflammation (6, 7).

Diazepam (DZ), which is a mixed-type benzodiazepine that can act on both central and peripheral benzodiazepine receptors, has been demonstrated to have an inhibitory effect on T cell function (8–12). However, the action on professional antigen-presenting cells such as macrophages (Mϕs) and dendritic cells (DCs) is not well characterized. In this work, we studied whether DZ impairs two undesired immune responses as septic shock and murine experimental autoimmune encephalomyelitis (EAE). The invasion of microbial pathogens into the bloodstream is characterized by a systemic pro-inflammatory response, which can lead to severe sepsis and septic shock. On the one hand, the innate immune cells as Mϕs, DCs, and neutrophils detect pathogen molecules by diverse receptors among which are the TLRs (13). Mϕ-derived cytokines, such as IL-6, TNF-α, and IL-1β, have been identified as central mediators in the pathogenesis of septic shock and the resultant multiple organ dysfunction syndrome that can lead to death (13). Here we described the bias from the classical activation of innate cells induced by LPS towards anti-inflammatory profiles when cultured with DZ, which prevented acute responses dependent on these cell populations. DCs are professional antigen-presenting cells and have a key role in initiating and regulating adaptive immune responses. Additionally, the DCs are also critical in suppressing immune responses and conserving peripheral tolerance through the generation of anergic and/or regulatory T cells and fine-tuning the response by changing the T-helper (Th1)/Th2/Th17 balance (14). Both immature and semi-mature DCs have been associated with an induction of tolerance through the generation of T_reg_ cells, the induction of apoptosis, or the anergy of autoreactive effector cells (15–17). In this work, we demonstrate the impaired LPS-induced activation when DC were co-treated with DZ and its inability to initiate Th1 and Th17 adaptive inflammatory responses.

On the other hand, EAE is a well-accepted model that mimics many of the clinical and pathological features of multiple sclerosis (MS). This pathology can be induced in C57BL/6 mice through immunization with MOG35–55 in CFA and produce monophasic or a chronic, sustained form of EAE. This model is characterized by a high induction of Th1 and Th17 autoimmune responses, mononuclear inflammatory infiltration and demyelination. Mϕ and CD4+ T cells are the main cell types in the inflammatory infiltrate (18). Here, we show that DZ not only prevented the onset of autoimmune responses, but more importantly, it also improved the clinical signs when it was administered therapeutically *in vivo*, once the disease was established, maintaining a low clinical score until 35 days post-immunization (dpi).

In summary, this work describes the immunomodulatory effects of Diazepam on the different stages of the immune response. DZ interferes with the activation of innate cells such as dendritic cells and macrophages induced by inflammatory stimuli and impairs the initiation and development of adaptive inflammatory responses (Th1 and Th17). Furthermore, DZ also favors the development of tolerogenic and anti-inflammatory responses such as Tregs or antigen-specific IL-10 producing cells. Our work contributes in a descriptive way to the knowledge of the immunomodulatory properties of this type of psychoactive drugs.

## Methods

### Bone Marrow-Derived Macrophage Generation and Culture

Cells were collected from femurs of C57BL/6 mice by flushing with 3 ml cold sterile PBS. The cell suspensions were passed through a sieve to remove large clumps, washed three times with sterile complete RPMI 1640 medium (Life Technologies, Grand Island, NY, USA) and 2 × 10^6^ cells/ml cultured with complete RPMI 1640 medium supplemented with 20 mM L-glutamine, 50 μg/ml gentamicin, and 10% heat-inactivated FCS in presence of 30% supernatant from macrophage colony-stimulating factor producing L929 fibrosarcoma cell line (20 ng/ml final concentration in the plate). After 3 days, the medium was changed, and at day 7 differentiated macrophages were collected and assessed by FACS analysis for F4/80 surface antigen expression using PE-labeled anti-F4/80 antibody (Caltag Laboratories, Buckingham, UK, clone BM8.1), indicating 95–98% of positive cells. The cells were cultured in a concentration 1.5 × 10^6^ cells/ml in 200 µl of final volume in 96-well plates at 37°C, with 5% CO_2_ for 18 h in complete medium with DZ (5 or 25 µM), LPS (1 μg/ml) or the combination of both. The concentration of nitrites was analyzed by a standard Griess reaction adapted to microplate as an indirect measurement of nitric oxide synthesis. The absorbance at 550 nm was obtained with a microplate reader model 680 (Bio-Rad Laboratories, Hercules, CA, USA). The data were referred to a standard curve of sodium nitrite (19).

### Western Blot

For immunodetection of iNOS, BMMϕ, which had been cultured (10^6^ cells/ml in a final volume of 500 µl) for 18 h in different conditions were harvested and lysed in Tris-HCl 10 mM pH 7.5, 1% SDS containing phosphatase and protease inhibitors. Equal amounts of protein (40 μg/lane) were separated in 10% SDS-polyacrylamide gel electrophoresis and transferred to a nitrocellulose membrane (GE Healthcare, Piscataway, NJ, USA). The INOS protein was revealed by immunoblotting using polyclonal anti-iNOS antibody (BD Biosciences, Franklin Lakes, NJ, USA, 610332) (20). Then, the membranes were washed thoroughly and incubated with IRDye 800CW anti-mouse IgG and IRDye 800CW anti-rabbit IgG antibodies for 1 h at room temperature. Immunodetection was performed with the Odyssey^®^ Infrared Imaging System (LICOR, Lincoln, NE, USA), and the immunoreactive protein bands were analyzed with the Gel-Pro Analyzer software (Media Cybernetics Inc., Bethesda, MD, USA).

### LPS-Induced Septic Shock

Six- to eight-week-old inbred female C57BL/6 (B6; H-2b) mice were purchased from Fundación José A. Balseiro (Centro de Energía Atómica Ezeiza, Buenos Aires, Argentina). All mice were kept under conventional conditions in the animal care facility of the Departamento de Bioquímica Clínica-CIBICI, Facultad de Ciencias Químicas (Universidad Nacional de Córdoba, Argentina) and treated in accordance with international and institutional guidelines for animal care. The experimental procedures were previously approved by the local Institutional Experimentation Animal Committee (Permit numbers 15-01-44195, and 832/2015). The number of animals used as well as their suffering was kept to the minimum possible needed to accomplish the goals of this study, n = 6/group. Three doses of 2 mg/kg DZ (Valium, Roche Internacional Limited, Montevideo, Uruguay) were i.p. administered in alternate days. After 12 h of the last DZ injection, each mouse was i.p. injected with 800 μg of LPS (*E. coli* 0111:B4; Sigma-Aldrich Co., St. Louis, Mo, USA). Untreated mice received PBS and LPS only. The onset of systemic clinical signs, including reduced mobility, lethargy, shivering, piloerection, and congested conjunctiva was recorded. Survival rate was determined every 6 h for 3 days post-septic shock induction, and then mice were euthanized by carbon dioxide overdose. To determine whether DZ treatment modulated the activation and cytokine profile of peritoneal cells (PCs), cells were obtained from untreated or DZ-treated mice after 12 h of the last DZ injection (n = 3/group) and then challenged with LPS *ex vivo* during 18 h, and the levels of cytokines were determined by ELISA.

### Flow Cytometry Analysis

After the treatments, the expression of surface molecules on DC or Mϕ was quantified by flow cytometry using FITC- or PE-conjugated antibodies (CD11c, F4/80, I-Ad, I-Ab, CD40), all purchased from BD Bioscience. Peritoneal cell populations were studied by flow cytometry using F4/80, CD11c, CD3, and CD19 antibodies (BD Bioscience). Samples were collected using a flow FACSCanto II (BD Bioscience, San Jose, CA, USA), and data were analyzed by Flowing Software2 (Turku Centre for Biotechnology, University of Turku, Finland).

### Cytokine Measurement

Cytokines were detected in culture supernatants using capture ELISA. IFN-*γ*, TNF-α, IL-10, IL-6, IL-17, and IL-12p70, all purchased from BD PharMingen (United States), were used as paired monoclonal antibodies in combination with recombinant cytokine standards. Assays were performed according to the manufacturer’s guidelines.

### DC Generation and Stimulation

DCs were generated as previously described (21). Briefly, bone marrow was collected from femurs of mice, and cells were seeded at 2 × 10^5^ cells/ml in 10 ml of RPMI 1640 complete medium supplemented with 2 mM L-glutamine, 7.5% culture supernatant from GM-CSF-producing J558 cells (final concentration 20 ng/ml), 10% endotoxin free FCS, and 50 μg/ml gentamicin. Cells fed on day 3 with complete RPMI medium containing GM-CSF and harvested on day 7 comprised 85% DC CD11c+ (16, 17). The direct effect of DZ on cell viability, activation status, production of cytokines, and activation of intracellular signaling pathways mediated by LPS was evaluated. In general, DCs differentiated from bone marrow were cultured in a concentration 1.5 × 10^6^ cells/ml in 200 µl of final volume in 96 well plates at 37°C, with 5% CO_2_ for 18 h in the presence of different concentrations of DZ (5, 25, 50 μM) with or without LPS (21). Cell viability was assessed with 3-(4,5-dimethylthiazol-2-yl)-2,5-diphenyltetrazolium bromide (MTT). Absorbance was recorded at 570 nm on a microplate reader (12).

### Allogeneic Mixed Lymphocyte Reaction

Differentiated DCs from C57BL6 mice were cultured for 18 h in the presence or absence of DZ with or without LPS. Collected cells were washed and co-cultured with allogenic splenocytes isolated from BALB/C mice, for 5 days at a 1:20 ratio (2 × 10^4^:42 × 10^5^) in 96-well plates. The supernatants were collected, and the cytokines levels were determined by ELISA (16).

### Study of *In Vivo* T_reg_ Cell Induction

Immature DCs from BALB/c mice were cultured for 18 h in the presence or absence of DZ, then were washed and 12 × 10^6^ cells were i.p. injected to C57BL/6 transgenic Foxp3EGFP mice. After 15 days, the animals were sacrificed and splenocytes collected. The population of CD4+CD25+FoxP3+ cells was analyzed by FACS canto II using anti-CD4 and anti-CD25 antibodies (eBioscience, San Diego, CA, USA) and FoxP3EGFP+ on the lymphocyte region by FACS canto II. Data were analyzed using Flowing software 2.5.0. (Turku Centre for Biotechnology, University of Turku, Finland).

### EAE Induction and Animal Treatment

Female C57BL/6 mice were purchased from the Jackson Laboratory (Bar Harbor, ME, USA). The encephalitogenic MOG35-55 peptide (M-E-V-G-W-Y-R-S-P-F-S-R-V-V-H-L-Y-R-N-G-K) was synthesized at the Johns Hopkins University Synthesis & Sequencing Core Facility (Baltimore, MD, USA) and purified to >99% *via* high-pressure liquid chromatography. Mice were immunized subcutaneously in two sites (left and right flanks) with 200 μg of MOG35-55 peptide that was emulsified in CFA (Sigma-Aldrich, St. Louis, MO, USA) containing 200 μg *Mycobacterium tuberculosis* (Difco Laboratories, Detroit, MI, USA). Mice received 250 ng pertussis toxin (Calbiochem, Gibbstown, NJ, USA) in 0.1 ml PBS by intraperitoneal injections at the time of immunization and 48 h later. Control mice were immunized with CFA followed by pertussis toxin. In order to evaluate the capability of DZ treatment to reduce established EAE, DZ was dissolved in PBS and administered i.p. (2 mg/kg/day) to mice challenged for the disease in alternate days from day 3 or when they reached score of 1. Mice were weighed and clinically scored daily. The onset of the disease correlates with weight loss, which might begin 1–2 days before EAE signs are visible and can be used as an indicator of disease activity. Clinical signs usually begin between days 9 and 14 and were scored as follows: 0, no deficit; 0.5, partial loss of tail tone or slightly abnormal gait; 1.0, complete tail paralysis or both partial loss of tail tone and mild hind limb weakness; 1.5, complete tail paralysis and mild hind limb weakness; 2.0: tail paralysis with moderate hind limb weakness; 2.5, no weight-bearing on hind limbs but with some leg movement; 3.0, complete hind limb paralysis with no residual movement; 3.5, hind limb paralysis with mild weakness in forelimbs; 4.0, complete quadriplegia but with some movement of head; 4.5, moribund; 5.0, dead. The mean clinical score was defined as the sum of the highest clinical score achieved by each mouse during the acute phase of the disease divided by the number of mice (22). Clinical scores of experimental groups were statistically analyzed by Mann–Whitney’s test, p <0.05. At 30 or 35 days dpi the animals were euthanized. The spleens and draining lymph nodes (DLNs) were aseptically removed and placed in ice-cold RPMI 1640 medium. Then the tissue was mechanically disrupted and single cell suspensions filtered through a 70 μm cell strainer (Nylon membrane BD, Becton Dickinson, Buenos Aires, Argentina). After washing with 50 ml Dulbecco’s PBS containing 2% FBS, cells were preserved in complete medium (RPMI 1640, 10% FBS, 40 mg/ml gentamicin). Then, 1.25 × 10^6^ cells/ml (250,000 cells/well) were cultured in triplicate in a volume of 200 μl in 96-well flat-bottom plates with MOG (10 µg/ml) or MOG/DZ for 96 h. Supernatants from cell cultures were harvested, and secreted levels of IFN-*γ*, IL-10, and IL-17 were determined using commercially available ELISA kits (BD Pharmigen, USA).

### Axon Counting

After 30 days post-immunization, mice were anesthetized with an overdose of isoflurane and perfused with 4% PFA and 2.5% glutaraldehyde. The spinal columns were postfixed overnight at 4°C, then C3–C5 cervical sections were isolated and further fixed with 1% osmium tetraoxide and embedded in Epoxipropyl ether of glycerol. Semi-thin 1 µm sections of C5 spinal segments were cut and stained with toluidine blue, and further imaged using a light microscope (Zeiss, Jena, Germany) equipped with a Leica LC200 video camera (Heerbrugg, Switzerland) ×40 magnification; 1.42 NA air objective were merged to reconstitute the dorsal column and adjacent areas. The axons in the medial dorsal column on the spinal cord at C5 were manually quantified. The dorsal columns are reproducibly damaged in this model of EAE (though the rostral–caudal level of damage was less predictable), and C5 was chosen to include ascending fibers from both the upper and lower limbs in the count (22). Using Fiji software an inverted triangular ROI was drawn within the gracile fasciculus: a line from the spinal canal to the dorsal surface was bisected to indicate the dorsal half of the column (height of the triangle) and a line perpendicular to this was drawn on both sides from the most medial part of the column to the ends of gracile fasciculus on the dorsal surface as described. Axon count was performed using cell counter plugin for Fiji. Only clearly visible, intact, myelinated axons >1 μm diameter were included.

### BV2 and MN-1 Co-Culture

The murine microglial cell line BV2 was a kind gift from Dr. Ivan Mascanfroni (Harvard Medical School, Center for Neurological Diseases, Bringham and Women’s Hospital, Boston, MA, USA). The cells were grown in DMEM supplemented with 10% heat-inactivated FCS, 2 mM glutamine and maintained at 37°C and 5% CO_2_.

Motoneuron-derived 1 (MN-1) cells, which are hybrids derived from embryonic mouse spinal cord motor neurons, were a kind gift from Dr. Pablo Lopez (Laboratory of Neurobiology, Instituto de Investigación Médica Mercedes y Martin Ferreyra, INIMEC-CONICET-Universidad Nacional de Córdoba, Argentina) and were routinely cultured in Dulbecco’s modified Eagle’s medium (DMEM), supplemented with 10% fetal bovine serum (FBS), 2 mM L-glutamine, in 5% CO_2_ at 37°C.

#### Co-culture

Briefly, BV2 microglial cells (1.5 × 10^6^/ml) were cultured in 12-well plates for 12 h with medium alone, LPS (1 µg/ml), DZ (25 µM) or the combination. Then, they were exhaustively washed and co-cultured with MN-1 cells that had been treated or not with DZ for 24 h at 5:1 ratio and were evaluated by flow cytometry for classical Annexin V/propidium iodide staining.

### Statistical Analysis

Statistical analyses were performed using the computer based GraphPad Prism Program V5.0 (GraphPad Software Inc., San Diego, CA, USA). Data were plotted as mean ± SD. A one-way ANOVA with Newman–Keuls multiple comparison test was used when three or more experimental groups were compared, and a paired t-test when two experimental groups were compared. P-values less than 0.05 were considered significant.

## Results

### DZ Diminishes LPS-Induced Classical Activation of BMMϕ

**Figure 1:**
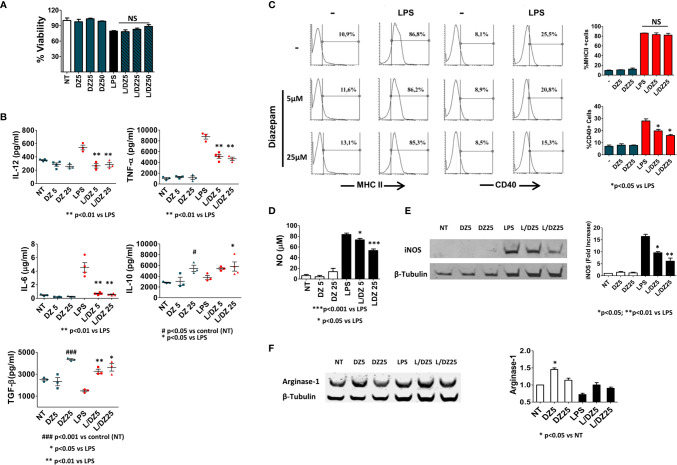
Diazepam (DZ) inhibits classical activation in macrophages cultured with LPS. C57BL/6 bone marrow-derived macrophages (4 × 10^5^ cells in 200 µl) were incubated in the presence of LPS (1 µg/ml), and DZ (5 and 25 μM) and evaluated after 18 h of culture **(A)** Percentage of cell viability was assessed with the MTT technique, **(B)** the supernatant was collected and IL-12p70, TNF-α, IL-6, IL-10, and TGF-β production was determined by ELISA, **(C)** MHCII and CD40 positive cells were assessed on F4/80+ macrophage population by FACS analysis, **(D)** Production of nitric oxide (NO) was evaluated by Griess reaction, and **(E, F)** levels of inducible nitric oxide synthase (iNOS) and Arginase-1 were determined by Western blot. Significant differences *vs.* LPS-treated cells are indicated by *p < 0.05, **p < 0.01, ***p < 0.001, and *vs.* nontreated cells (NT) by ^#^ *p* < 0.05 and ^###^ *p* < 0.001. Non-significant values are expressed as NS. The graphs are representative of at least three independent experiments.

Since Mϕs are one of the major cells involved in inflammation mediated by innate immunity, we evaluated the direct effect exerted by DZ on this cell population. For that, BMMϕs were cultured with medium alone, LPS (1 μg/ml), DZ (5, 25 μM) or the combinations of LPS and DZ. After 18 h, the supernatants were collected to measure cytokine secretion, and viability was evaluated by MTT test. The presence of DZ did not affect cell viability in any concentration evaluated, either in the presence or in the absence of LPS (**Figure 1A**). However, the presence of benzodiazepine was able to inhibit the LPS-induced secretion of pro-inflammatory cytokines as IL-12p70, TNF, and IL-6 (**Figure 1B**). Furthermore, macrophage treatment with DZ at 25 µM augmented IL-10 secretion, while in the presence of LPS, macrophage treatment with DZ significantly increased IL-10 secretion at only 5 µM (**Figure 1B**). Moreover, DZ increased the secreted levels of TGF-β in immature macrophages at 25 µM only, while in the presence of LPS, an increase was observed in the two concentrations of DZ tested (**Figure 1B**). On the other hand, DZ reduced the LPS-induced CD40 expression in a dose dependent manner but did not modify MHCII+ cell percentage, which is consistent with a semi-mature state (**Figure 1C**). In addition to pro-inflammatory cytokine secretion, the levels of NO release and iNOS were studied as classical activation markers of Mϕ cells. We observed that DZ partially inhibited the LPS-induced iNOS expression as well as NO secretion in a dose dependent manner (**Figures 1D, E**). Since Arginase-1 is an alternative activation marker in macrophages, we wondered if DZ was capable of modulating this enzyme. We observed that DZ subtly increased Arginase-1 levels at 5 µM concentration, although it only did so significantly in the absence of LPS (**Figure 1F**). Altogether, these results indicate that DZ interferes in the classical activation of BMMϕ induced by LPS promoting a semi-mature state with increased levels of anti-inflammatory cytokines.

### DZ Decreases the Susceptibility to LPS-Induced Endotoxic Shock

**Figure 2:**
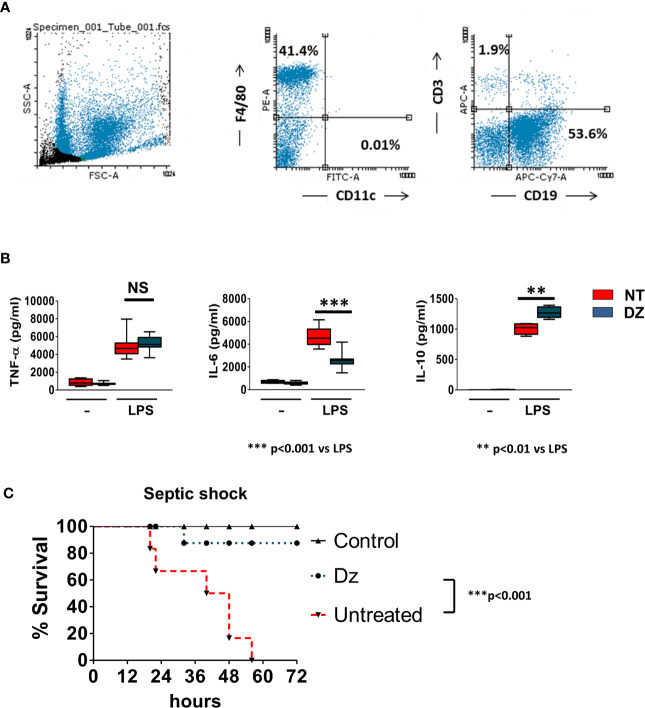
Diazepam (DZ) protects mice from LPS-induced septic shock. **(A)** Peritoneal cells (PCs) from untreated control mice were obtained, stained with F4/80 (PE), CD11c (FITC), CD3 (APC), and CD19 (APC-Cy7) and then were evaluated by flow cytometry. **(B)** DZ-treatment modulates the activation and cytokine profile of peritoneal cells (PCs). PCs from untreated or DZ-treated mice were obtained after 12 h of the last DZ injection and challenged in *vitro* with LPS during 18 h. TNF-α, IL-6, and IL-10 production by was measured in supernatants by ELISA. Data are representative of two independent experiments (n = 3). *p* was calculated by Student’s *t*-test for grouped samples and the probability of significance with respect to the untreated control is indicated by NS: not significant, **p < 0.01, ***p < 0.001. **(C)** C57BL/6 mice were treated with three doses of DZ (2 mg/kg) or PBS administered in alternate days, and 12 h after the last treatment, the mice were i.p. injected with 800 μg of LPS. Control non-treated unchallenged mice (▲), PBS-treated LPS-challenged mice (▼), DZ-treated LPS-challenged mice (●). Data are representative of two independent experiments (n = 6). Statistical analysis was performed using log-rank test. ***p < 0.001 between PBS-treated and DZ-treated LPS-challenged mice. Non-significant values are expressed as NS.

Taking into account that DZ decreased the *in vitro* LPS-induced activation of macrophages and that they are one of the main populations on the total of peritoneal cells (PCs) (**Figure 2A**), we decided to study whether the *in vivo* administration of DZ was capable of conditioning the inflammatory response of PCs to challenge with LPS. For this objective, three doses of DZ (2 mg/kg) were administrated (i.p.) in alternate days, and then peritoneal cells obtained from PBS-or DZ-treated mice were washed and stimulated *in vitro* with LPS. Although no changes were observed in the TNF-α production, PCs from DZ-treated mice had a reduced IL-6 secretion and an increased IL-10 production compared to PCs from PBS-treated mice when cultured with LPS (**Figure 2B**). The inverted IL-6/IL-10 secretion ratio induced by *in vitro* LPS-stimulated PCs suggests that DZ modulates the innate immunity, favoring an anti-inflammatory profile commanded by IL-10 secretion.

After establishing that the administration of DZ conditioned the response of the PC to LPS, we decided to challenge the mice, treated with DZ as indicated above, to the i.p. injection with LPS (800 µg), and survival percentage was scored until 72 h post-induction. We observed that DZ-treated mice showed a decreased score of systemic clinical signs, including reduced mobility, lethargy, shivering, piloerection and/or congested conjunctiva in contrast to untreated mice (data not shown). More importantly, DZ treatment significantly increased the survival of the LPS-challenged mice (87%) at 72 h compared to those that were not treated (**Figure 2C**).

These data, together with those obtained *in vitro* and shown in **Figure 1**, suggest that treatment with DZ disables the classical activation of innate immune cells induced by LPS, inhibiting the secretion of inflammatory cytokines and preventing exacerbated inflammatory responses *in vivo* such as those that are developed in the septic shock model.

### DZ Treatment Induces Semi-Mature DC in the Presence of LPS

**Figure 3:**
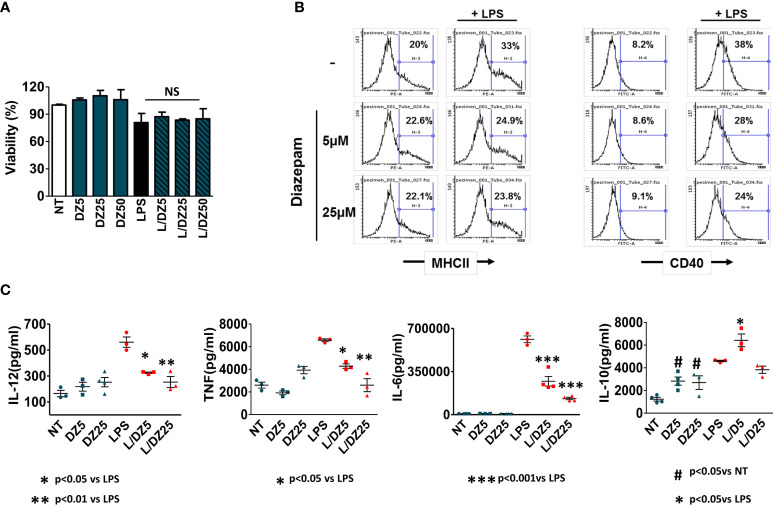
Diazepam (DZ) has an inhibitory effect on LPS-induced dendritic cell (DC) activation. Cells were isolated from bone marrow of C57BL/6 mice and differentiated into DCs. Immature DCs (1.5 × 10^6^ cells/ml in 200 µl final volume) were incubated in the presence of LPS (1 µg/ml), DZ (5 and 25 μM) and evaluated after 18 h of culture. **(A)** Cell viability was evaluated with diazepam concentrations up to 50 μM. However concentrations of 5 and 25 μM were chosen as low and high respectively. **(B)** MHCII positive cells and costimulatory CD40 expression were determined using specific antibodies and analyzed by flow cytometry, **(C)** Cytokine production (IL-12, TNF-α, IL-6, IL-10) was evaluated by ELISA in supernatants. Data are representative of three independent experiments. Significant differences *vs.* LPS-treated cells are indicated by *p < 0.05, **p < 0.01, ***p < 0.001 and *vs.* nontreated cells by ^#^ *p* < 0.05. Non-significant values are expressed as NS.

Since DCs play a key role within innate immunity due to their unique capacity to induce activation of naïve T cells, we decided to study the DZ effect on the maturation and activation of these cells. Bone marrow-derived DCs were cultured during 18 h with or without LPS (1 μg/ml) in the absence or presence of DZ (5 and 25 μM). DZ treatment did not modify cell viability of immature or LPS-matured DC until 50 μM (**Figure 3A**). However, DZ-treated DC exhibited low expression of cell-surface molecules upon LPS stimulation (MHCII and CD40) at both tested concentrations (**Figure 3B**). In a similar way to BMMϕ, the incubation of DC in the presence of DZ decreased LPS-induced secretion of IL-12p70, TNF-α, and IL-6 while basal levels were not significantly altered (**Figure 3C**). Furthermore, DZ at the concentration of 5 µM significantly increased the secretion of IL-10 in the presence of LPS, while in its absence an increase was observed in both concentrations of DZ tested (**Figure 3C**). These results suggest that similarly to macrophages, DZ is capable of inducing and promoting DC towards a semi-mature regulatory phenotype.

### DZ-Treated DC Induces a Decreased Inflammatory Allogeneic Response

**Figure 4:**
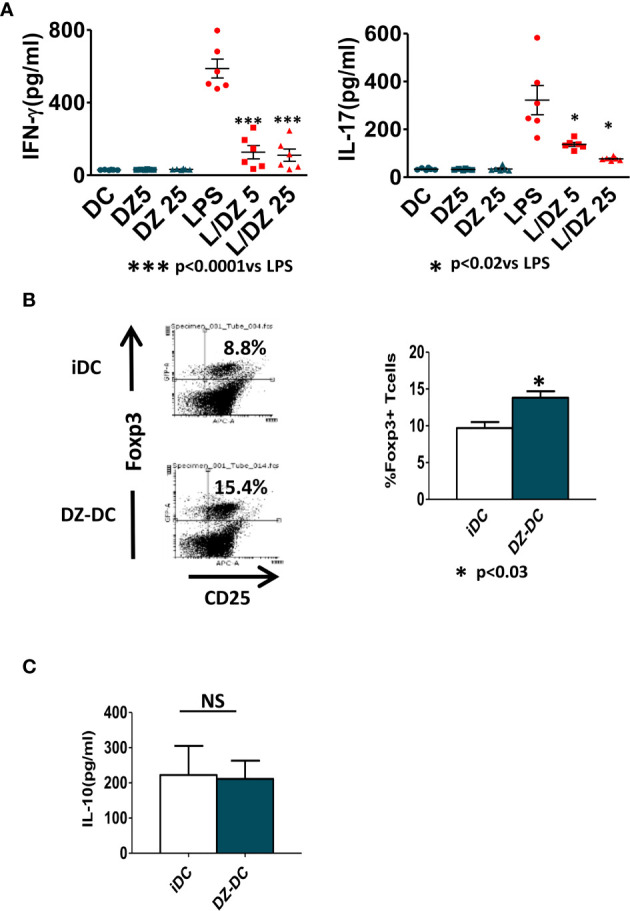
Impaired ability of Diazepam (DZ)-treated dendritic cells (DCs) to initiate allogeneic inflammatory responses. **(A)** C57BL/6 bone marrow-derived DCs (1.5 × 10^6^/ml) were pre-incubated with LPS and DZ for 18 h, washed, and co-cultured with allogeneic splenocytes (SP) from BALB/c mice during 5 days at DC : SP relation of 1:20. IFN-*γ* and IL17 levels were determined by ELISA in supernatants. **(B)** Balb/c bone marrow-derived DCs (1 × 10^6^) untreated or treated with DZ (25 µM) were injected i.p. in C57BL/6-Foxp3-GFP reporter mice (n = 3). At day 15 post-injection, the mice were sacrificed, and the determination of CD4+CD25+GFP+cells on spleen cell population was evaluated by flow cytometry. **(C)** Splenocytes from iDC and DZ-DC treated mice were cultured for 24 h in the presence of PMA, and IL-10 production was evaluated by ELISA test on culture supernatants. These data are representative of at least three experiments. Significant differences are indicated by *p < 0.05, ***p < 0.001. Non-significant values are expressed as NS.

To investigate how DZ treatment of immature or LPS-matured DC influenced the onset of inflammatory adaptive response, DCs were cultured for 18 h with medium alone, DZ (5 and 25 μM), LPS (1 μg/ml), and the combination of DZ and LPS (L/DZ5 and L/DZ25). Then, the cells were washed and co-cultured with allogeneic splenocytes during 5 days. While no significant differences were detected in the DZ-treated DCs compared to immature non-treated DCs, DZ in combination with LPS-stimulated DCs was less able to induce IFN-*γ* and IL-17 secretion in the allogeneic co-culture in contrast to LPS-DC condition (**Figure 4A**). These data confirm that a defective LPS-induced DC maturation occurred in the presence of DZ. Since the manipulation of immature DCs has been used to diminish the rejected responses against allogeneic transplants, and herein we did not observe differences between non-treated or DZ-treated immature DCs, we decided to investigate whether DZ treatment of DCs conferred any advantage compared to untreated DCs to generate regulatory mechanisms. For this reason, we injected allogeneic immature DCs pre-incubated for 18 h in the absence (iDC) or presence of DZ (DZ-DC) in Foxp3EGFP C57BL/6 transgenic mice. After 15 days, the animals were sacrificed, and the presence of CD4+CD25+GFP+ cells was assessed in the spleen. Although treatment with immature DCs showed an enhanced percentage of T_reg_ with respect to untreated mice (data not shown), we observed a significant increase in the percentage of Foxp3 T_reg_ in mice that received DZ-treated DC compared to those that received immature DCs (**Figure 4B**). However, no differences in IL-10 production were observed in cultures of splenocytes from iDC or DZ-DC treated mice (**Figure 4C**). Shortly, these data suggest that DZ prints immunomodulatory properties on both immature and matured DCs to bias inflammatory adaptive responses to a tolerogenic response.

### Diazepam Treatment Prevented EAE Severity and Decreased the Specific Inflammatory Adaptive Response Against MOG *In Vitro*

**Figure 5:**
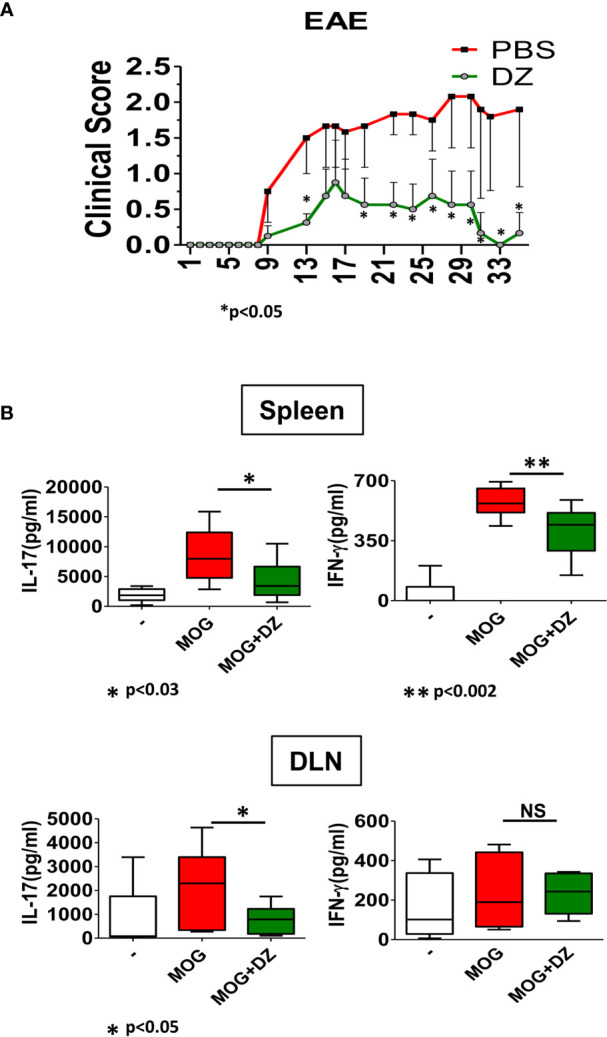
Diazepam treatment reduced the EAE clinical signs and MOG-specific cytokine production. **(A)** DZ or vehicle was i.p. administered to mice challenged for the disease in alternate days from day 3 p.i. and then were scored daily. Clinical scores of experimental groups were statistically analyzed by Mann–Whitney test, p < 0.05 **(B)** Production of IL-17 and IFN-*γ* by cells isolated from spleen and draining lymph nodes (DLN) from nontreated EAE animals at 30 dpi and incubated in the presence of MOG (10 µg/ml) or MOG/DZ was determined. These data are representative of at least 2 experiments. Significant differences of MOG/DZ- *vs.* MOG-treated cells are indicated by *p < 0.05 and **p < 0.01. Non-significant values are expressed as NS.

Because DZ induced defective innate immunity activation and a diminished ability of DZ-treated DC to initiate an inflammatory adaptive response, we hypothesized that the administration of DZ at the beginning of EAE induction could prevent disease progression. For that, DZ was administered intraperitoneally in alternate days from 3 to 33 dpi, and clinical signs were scored throughout the entire treatment. We observed that DZ treatment from inductive phase of EAE reduced the severity of the disease as demonstrated by the low clinical score and maintaining the average score below 1 (**Figure 5A**). Then, in order to know if DZ could decrease MOG-specific inflammatory response on cells from EAE primed mice *in vitro*, cells isolated from spleen and draining lymph nodes (DLN) of non-treated EAE animals at 35 dpi were incubated in the presence of MOG with or without DZ. The DZ addition reduced the MOG-specific production of IL-17 on splenocytes and DLN cells, although the release of IFN-*γ* only decreased in splenocytes (**Figure 5B**). These results suggest that DZ was also able to modulate the inflammatory adaptive responses *in vivo* at its onset when the primary response to the antigen occurred. Furthermore, there was a diminished response to MOG of splenocytes from MOG primed mice when cultured in the presence of DZ.

### Therapeutic DZ Administration Ameliorates the Severity of EAE

**Figure 6:**
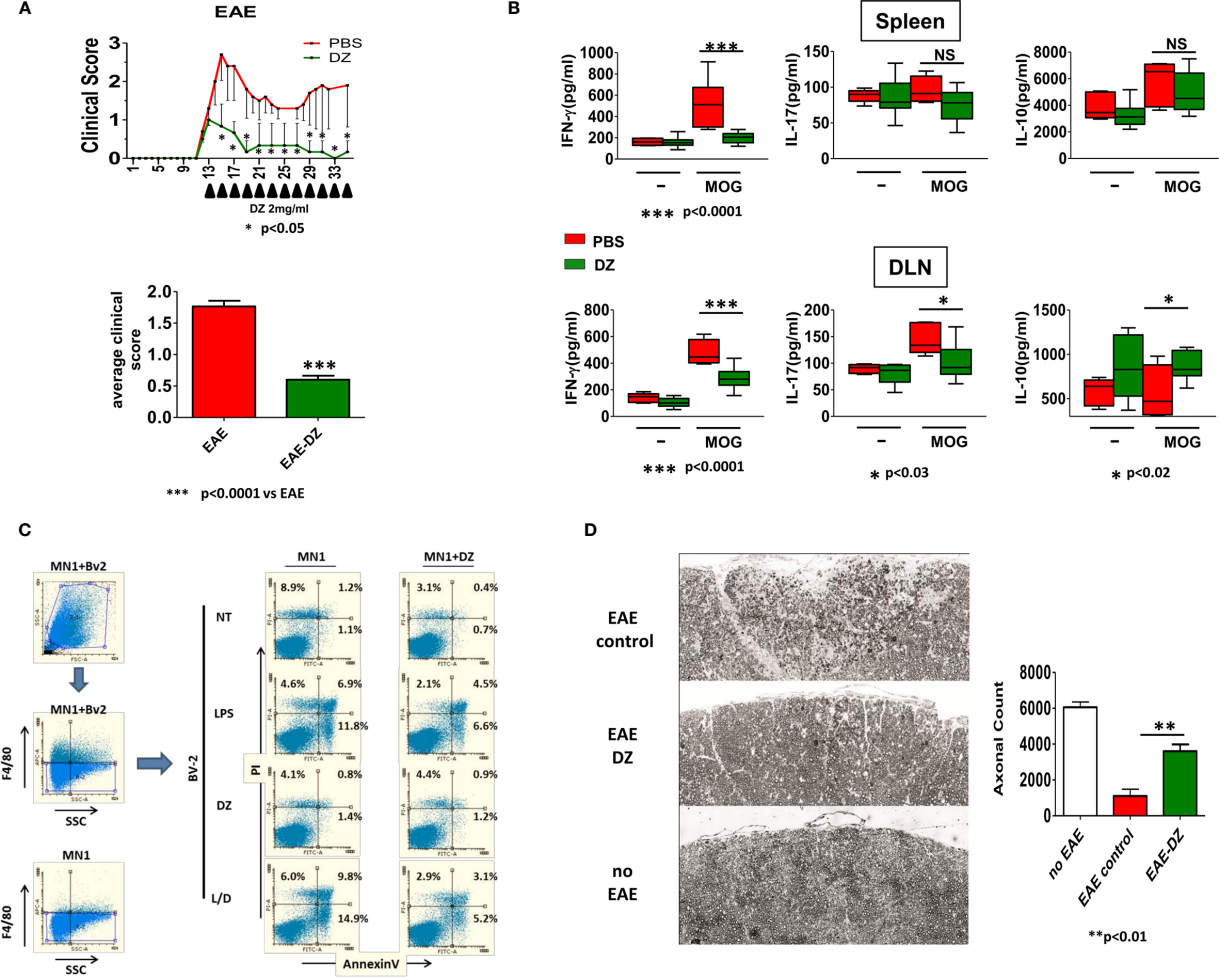
Therapeutic DZ administration reduces the severity of EAE. DZ was i.p. administered to mice challenged for the disease in alternate days when they reached a score of 1. **(A)** Clinical score was evaluated daily. Clinical scores of experimental groups were statistically analyzed by Mann–Whitney test, p < 0.05. **(B)** Cells isolated at 35 dpi from spleen and draining lymph nodes (DLN) from EAE animals, treated or untreated with DZ, were re-stimulated in *vitro* in the presence of MOG (10 µg/ml) and IFN-*γ*, IL-17, and IL-10 production was determined by ELISA. **(C)** BV-2 microglial cells were cultured with medium, LPS (1 µg/ml), DZ (25 µM) or combination LPS plus DZ, for 18 h, then were co-cultured with untreated or DZ-treated MN-1 cells. Apoptosis and death of F4/80 negative cells were analyzed by flow cytometry. **(D)** DZ was i.p. administered to mice challenged for the disease in alternate days from day 7. At day 35 p.i. mice were sacrificed, and the axons in the medial dorsal column on the spinal cord at C5 were manually quantified (n = 6). Significant differences of cells from DZ- *vs.* non-treated EAE animals are indicated by **p < 0.01. Non-significant values are expressed as NS.

In order to evaluate the capability of DZ treatment to reduce established EAE, MOG-induced EAE mice were treated in alternate days with DZ when they achieved the clinical score of 1. The i.p. DZ injection significantly reduced the severity of EAE, reaching a maximum clinical score of 1.5 and retaining a score below 1 during the rest of the treatment (**Figure 6A**). In comparison non-treated EAE animals reached a maximum of three averaging clinical score values between 1.5 and 2 until 35 dpi (average clinical score: EAE = 1.767 ± 0.08884 *vs.* EAE-DZ = 0.6000 ± 0.06380) (**Figure 6A**). In addition, cells isolated at 35 dpi from spleen and DLN from EAE animals, treated or not with DZ, were re-stimulated *in vitro* in the presence of MOG for 96 h. As observed, DLN cells isolated from DZ-treated EAE animals released lower levels of Th1 and Th17 inflammatory cytokines (IFN-*γ* and IL-17) and higher levels of the anti-inflammatory cytokine IL-10 than untreated mice (**Figure 6B**). In spleen cells, DZ-treated EAE animals only showed lower levels of IL-17 with respect to untreated mice. Altogether, these data suggest that DZ is a potent immunomodulatory drug able to control undesired immune responses in progress as autoimmune diseases, decreasing antigen specific inflammatory cytokines.

### DZ Prevented Neuronal Death Induced by Activated Microglial Cells *In Vitro* and Axonal Damage in EAE Model

Since glutamate excitotoxicity has been related to neuronal and axonal damage during EAE and considering that activated microglia is one of the main sources of glutamate and other neurotoxins, we evaluated the apoptosis and death of neurons induced by LPS-activated microglial cells. In this sense, BV2-immortalized murine microglial cells were treated with LPS (1 µg/ml), DZ (25 µM), or the combination during 18 h. Then, they were exhaustively washed and co-cultured with mouse spinal cord motor neuron hybrid cell line MN-1, untreated or previously DZ-treated, during 24 h and evaluated by flow cytometry. We observed that LPS-activated BV2 microglia increased the percentage of early and later apoptosis in F4/80 negative MN-1 cells (**Figure 6C**) compared to untreated BV2 cells. Although DZ treatment was able to diminish LPS-induced BV2 activation (L/DZ), as observed by low levels of TNF (data not shown), these cells induced the highest apoptosis levels on neuronal cells. However, MN-1 neurons that were previously cultured with DZ for 24 h showed to be less sensitive to apoptosis and death induced by LPS-activated BV2 (**Figure 6C**). Even more, DZ-treated MN-1 showed lower values of apoptosis and cell death when cultured with BV2 microglial cells in all treatments compared with untreated MN-1 (**Figure 6C**).

According to these observations, we investigated whether DZ was able to prevent the axonal damage previously described in the murine EAE (22). In this sense, it has been demonstrated that mice challenged to EAE immunization showed axonal damage and demyelination from day 7 post immunization. Mice were treated from day 7 to day 30 with DZ or PBS. On day 35 mice were anesthetized and perfused with 10 ml of PBS followed by 10 ml prepared 4% (w/v) para-formaldehyde in PBS and collected C5 from the spinal cord for axon counting. We observed a drastic reduction in the number of myelinated axons in mice that were immunized and did not receive treatment with DZ (approximately 82% reduction compared to non-EAE control mice), while the animals that received it maintained three times more intact myelin fibers, which represents almost 60% of the existing fibers in non-EAE mice (**Figure 6D**). These results suggest that DZ diminishes neuronal damage decreasing the harmful effects of the disease.

## Conclusion

DZ is a powerful immunosuppressant capable of disarming inflammatory immune responses, inhibiting the onset either by disabling antigen presentation as well as the secondary signals necessary for the correct priming of adaptive immune cells and their subsequent polarization towards inflammatory profiles. It also prevents the progression of established inflammatory responses as observed in the therapeutic treatment of EAE mice. This treatment seems to provide advantages to contain exacerbated immune responses that are harmful to the body

## Discussion

Benzodiazepines are widely used psychoactive drugs that share similar pharmacological properties, such as sedative, hypnotic (sleep-inducing), anxiolytic, and anticonvulsive action and are widely used adjuncts to anesthesia to induce central muscle relaxation and amnesia (1). Previous studies have shown that an endogenous inhibitory GABAergic system is present in cells of the immune system as DC and monocytes and could regulate immune responses (23–25). It has been demonstrated that DC is able to synthesize and release the classical neurotransmitter GABA, and it also expresses GABA receptor subunits (26). The autocrine/paracrine influence produces immunomodulatory effects on this cell population and decreases innate and adaptive inflammatory responses initiated by these cells (27). On the other hand, TSPO ligands showed immunosuppressive properties, decreasing inflammatory cytokines by microglial cells stimulated with LPS (28). In agreement with this, the production of IFN‐*γ* by human memory and naïve CD4+ T cells was inhibited by Diazepam suppressing the activity of the transcription factors as T‐bet and STATs (9). DZ is a benzodiazepine capable of binding both receptors. In our study, we demonstrated that DZ has modulatory properties on the immune system, exerting a suppressive effect on the different stages of the immune response. First, we showed that DZ prevents the LPS-induced classical maturation of Mϕ and induced a semi-mature phenotype in this cell type, with increased IL-10 and TGF-*β* secretion, normal expression of MHCII but reduced expression of CD40 and pro-inflammatory cytokines. Since Mϕs, together with neutrophils, are the major responsible immune cells for acute inflammatory responses dependent on innate immunity (29, 30), we hypothesize that DZ could prevent such responses. Innate immunity plays a key role in the development of LPS-endotoxic shock and thus represents a good model for the study of the modulatory capacity of DZ on innate immunity *in vivo* (31). Here, we showed that DZ treatment was able to restrain this inflammatory response, encouraging a greater survival of mice challenged with large doses of LPS. Several studies have elucidated many different pathophysiologic processes involved in sepsis and have revealed an important regulatory role of pro- and anti-inflammatory cytokines in disease progression (13). In agreement with these results, we demonstrate here that this drug modulates LPS-induced PC activation by inhibiting IL-6 secretion and favoring IL-10 production, suggesting that DZ changes the manner in which these cells respond to LPS. IL-10 has been demonstrated to be an important M2 cytokine, and it controls the onset of irreversible septic shock due to anti-inflammatory properties (32, 33). However, the alteration of the immune response could also be due to the increase of corticosterone induced by DZ as previously described (34).

On the other hand, DCs are the cells responsible for connecting the innate and adaptive immune systems, presenting antigens to both naive and memory T cells and initiating appropriate immune responses (35). There is a close correlation between the activation state achieved by these cells and the adaptive response to be assembled. Thus, increased activation of DC with high co-stimulatory molecule expression and IL-12 production favors a Th1 profile, whereas TGF-*β* plus IL-6 develops a Th17 profile. Both inflammatory profiles are protective in infections caused by bacteria or fungi, but may be undesirable in circumstances such as organ transplants or autoimmune diseases, in which it is required to induce tolerogenic responses. For these reasons, we evaluated whether DZ was able to modulate DCs to a regulatory phenotype. DZ treatment not only decreased the LPS-induced DC activation, but it also impaired cell ability to initiate Th1 and Th17 allogeneic inflammatory responses. Even more, DZ was capable of printing a regulatory state on immature non-LPS-treated DCs, which favored an increase in the population of Foxp3+ T_reg_ cells. These results suggest that DZ is able to induce mechanisms that bias the classical activation of the innate cells, which is induced by inflammatory stimuli, to semi-mature phenotype that conditioned the adaptive responses to regulatory profiles.

EAE model is characterized by a high induction of Th1 and Th17 autoimmune responses, mononuclear inflammatory infiltration to CNS and demyelination. Mϕ and CD4+ T cells are the main cell types in the inflammatory infiltrate (18, 36) while migratory myeloid DC has been involved in the pathogenesis of this disease as an antigen presenting cell necessary to initiate autoimmune responses (37). Since DZ impairs the ability of DC to initiate inflammatory responses *in vitro*, we wanted to know whether this drug was able to prevent the initiation of such responses *in vivo* in an EAE murine model. DZ treatment from day 3 in MOG-induced EAE mice prevented the exacerbation of the clinical signs, maintaining a low score throughout the evaluated period. Although DC and macrophages as well as activation states were not evaluated *in vivo*, we recognize the key role these cells play in both the initiation and development of the disease (38–40). Moreover, the goal of this work was to evaluate the ability of DZ to suppress the different phases of the immune responses. We suggest that DZ could affect not only the maturation of APCs and their ability to initiate adaptive responses, but it could also have an effect on the expansion and proliferation of antigen-specific T cells as we have previously shown (12). Therefore, herein we demonstrated that DZ suppressed established inflammatory responses *in vitro*. Both, lymph node cells and splenocytes from EAE-mice were less able to respond to re-stimulation with MOG in the presence of DZ. Although this could also be due to the inability of APCs to present antigens, we hypothesize that DZ also exerts a direct effect on lymphocytes and their ability to be activated. In this sense we previously demonstrated that DZ has an effect on inhibiting lymphocyte activation *in vitro* (11, 12). These results suggested that DZ could be an alternative treatment for autoimmune disorders as MS. In order to demonstrate this hypothesis, we administered DZ therapeutically to mice that achieved clinical score of 1 in the EAE model. Surprisingly, this treatment improved the clinical signs and impaired the disease progression compared to non-treated mice to the point of almost reversing the disease completely in all treated animals. Moreover, splenocytes and DLN cells from DZ-treated mice showed an increased anti-inflammatory response commanded by IL-10 and less capability to release specific IL-17 and IFN-*γ* to MOG re-stimulation. A very interesting point of our work was to demonstrate the ability of DZ to promote the production of IL-10 on *in vitro* and *in vivo* experiments. IL-10 is a very important cytokine in the control of EAE, and it has been shown that mice that overexpress it are resistant to the induction of EAE, while IL-10 KO mice show an increased severity of the disease (41, 42). Among some of the mechanisms exerted by IL-10 are the capability to reduce the production of inflammatory cytokines by T cells and prevent prolonged contact between them and antigen-presenting cells, favoring regulatory responses. Furthermore, it has been shown that IL-10 produced by Tr1 regulatory T cells in an EAE model prevents the inflammatory phenotype of astrocytes and microglia, thus decreasing demyelination and recruitment of monocytes in the CNS (43). In agreement with these findings, we observed that therapeutic treatment of EAE-mice with DZ was able to control the specific inflammatory response against MOG and almost completely reversed the clinical signs of the disease. More important, a higher IL-10 secretion and a reduced IL-17 production in draining lymph nodes seemed to correspond with less severity in the development of clinical signs. Although we only treated those mice that showed clinical signs, we also observed that draining lymph node cells but not splenocytes from non-sick EAE-mice showed higher IL-10 release and lower IL-17 production than mice with clinical signs, when they were re-stimulated with MOG (data unpublished). In addition, DZ treatment also prevented axonal damage and maintained a high percentage of myelinated sensitive fibers. However, not only did we show effects on immune cells but we also observed a refractory response of MN-1 neuronal cells to apoptosis and death induced by LPS-activated microglial cells. It has been proposed that IL-10 exerts neuroprotective effects by blocking the glutamate-mediated induction of caspase-3 and NF-κB activation (44), which may contribute to improving clinical signs and preserving sensitive fibers. In accordance with these data as a whole, we hypothesized that the increase in IL-10 in mice treated with DZ could be responsible, at least in part, for the protective effects shown by this drug. We suggest that the ability of DZ to induce regulatory mechanisms such as IL-10 and TGF-β secretion or generation and expansion of Tregs creates an anti-inflammatory environment capable of containing and controlling exacerbated immune responses. Thus, DZ decreases ongoing inflammatory immune responses and impairs the presentation of novel antigens as it occurs in MS disease developing regulatory mechanisms that could control the new inflammatory foci.

## Conclusion

Altogether, these results show that DZ possesses immunosuppressive properties which decrease and control undesirable responses from innate and adaptive immunity. In the future, further studies should be carried out to determine the role of NMDA and AMPA receptors in the neuroprotective effects exerted by DZ. We propose DZ as an alternative preventive treatment, able to diminish exacerbated immune responses.

## Data Availability Statement

The original contributions presented in the study are included in the article/supplementary material. Further inquiries can be directed to the corresponding autor.

## Ethics Statement

The experimental procedures were previously approved by the local Institutional Experimentation Animal Committee (Permit numbers 15-01-44195, and 832/2015).

## Author Contributions

CF: Design and experimental execution, analysis of experimental results, and manuscript writing. NH: Western blot and viability assays. AV and PL: Axonal fiber count tests and histologic analysis. AZ and GG: Experimental design, manuscript correction, figure design, and flow cytometry assays. LC, CM, and GR: Experimental design, manuscript correction, figure design, and financial support. All authors contributed to the article and approved the submitted version.

## Funding

This work was supported in part by Consejo de Investigaciones Científicas y Técnicas [PIP 112-201101-00501] [PIP-2017-2019], Agencia Nacional de Promoción Científica y Tecnológica [Préstamo BID, PICT2011-0799] [PICT2017-4595], Florencio Fiorini Foundation, PUE2016-CIQUIBIC-CONICET and Secretaría de Ciencia y Tecnología de la Universidad Nacional de Córdoba, Argentina.

## Conflict of Interest

The authors declare that the research was conducted in the absence of any commercial or financial relationships that could be construed as a potential conflict of interest.

## Abbreviations

BMMϕ, bone marrow-derived macrophages; CNS, central nervous system; DC, dendritic cell; DLN, draining lymph node; dpi, days post-immunization; DZ, Diazepam; GABA, γ-aminobutyric acid; iNOS, cytokine-inducible nitric oxide synthase; Mϕ, macrophages; MLR, mixed leukocyte reaction assay; MoDC, monocyte-derived dendritic cells; PC, peritoneal cells.
